# Age at Transition from Pediatric to Adult Care Has No Relationship with Mortality for Childhood-Onset Type 1 Diabetes in Japan: Diabetes Epidemiology Research International (DERI) Mortality Study

**DOI:** 10.1371/journal.pone.0150720

**Published:** 2016-03-03

**Authors:** Yoshiko Onda, Rimei Nishimura, Aya Morimoto, Hironari Sano, Kazunori Utsunomiya, Naoko Tajima, The Diabetes Epidemiology Research International (DERI) Mortality Study Group

**Affiliations:** 1 Division of Diabetes, Metabolism and Endocrinology, Department of Internal Medicine, Jikei University School of Medicine, Tokyo, Japan; 2 Graduate School of Public Health, University of Pittsburgh, Pittsburgh, Pennsylvania, United States of America; 3 Department of Internal Medicine, Morimoto Hospital, Tokyo, Japan; 4 Jikei University School of Medicine, Tokyo, Japan; University of Illinois at Chicago, UNITED STATES

## Abstract

**Objective:**

To follow up Japanese patients with type 1 diabetes for a maximum of 40 years to examine when they transitioned from pediatric care to adult care and to explore whether the attending physician, i.e., pediatrician or internist, was associated with prognosis.

**Methods:**

Participants consisted of 1,299 patients who had been diagnosed as having type 1 diabetes at less than 15 years old between 1965 and 1979 identified through two nationwide surveys. Patients were classified as having received either pediatric care or adult care at the age of 15 and 30, and were compared for differences in mortality associated with the attending physician.

**Results:**

The attending physicians were confirmed for a total of 1,093 patients at the age of 15. Of these patients, 43.8% and 40.3% received pediatric care and adult care, respectively. Of the 569 patients receiving pediatric care, 74.2%, 56.6%, 53.4%, and 51.3% continued with pediatric care at 20, 30, 40, and 50 years old, respectively. The attending physicians (pediatrician or internist) at the age of 15 and 30 had no significant impact on their survival (*P* = 0. 892, 0.411, respectively).

**Conclusions:**

More than half of the patients who had received pediatric care at the age of 15 continued to receive pediatric care even after the age of 30, suggesting that their transition was far from smooth, while the attending physician at the age of both 15 and 30 was not a prognostic factor for mortality. Thus, the timing for transition to adult care in these patients has no relationship with mortality in Japan.

## Introduction

Despite recent advances in diabetes care, the mortality of childhood-onset type 1 diabetes is much higher than that seen in the general population [1–3]. A likely explanation for this excess mortality among patients with childhood-onset type 1 diabetes is that patients with type 1 diabetes in adolescence to emerging adulthood tend to show suboptimal glycemic control, which places them at risk of future diabetic complications [4–8].

Additionally, during this period, individuals in their teens to early twenties, including healthy people, experience significant physical, emotional, psychological, and social changes [5, 9]. Moreover, patients with type 1 diabetes have to go through these changes while living with diabetes, which can make things more difficult.

In addition to undergoing many stressful changes in their lives, such as seeking higher education, establishing careers and romantic/marital relationships, and leaving home, they are in a transition from pediatric to adult care, which is itself a factor contributing to the worsening of glycemic control [9, 10].

While parents and guardians oversee a large aspect of pediatric care, patients with childhood-onset type 1 diabetes are expected to face formidable challenges in taking over responsibility for their own self-care during their transition from pediatric to adult care. Furthermore, adult care is generally less forgiving of the behavioral and developmental struggles of the patients, and this can be unsettling to some patients [11, 12]. Such gaps between pediatric and adult care [13] have been associated with suboptimal medical supervision [14, 15], deteriorating glycemic control, progression of chronic diabetic complications, and emergence of acute fatal diabetic complications in these patients, leading to early mortality in some cases [9, 10]. Again, childhood-onset type 1 diabetic patients may be required to face further challenges than their own self-care, which include coping with the development of secondary sex characteristics during adolescence and diabetic complications such as lifestyle-related diseases from adolescence onwards. Thus, clinical practice for patients with type 1 diabetes entails enlisting the expertise of both pediatricians and internists as the patients move from childhood to adulthood, and their attending physician’s specialty, pediatrics or internal medicine, may affect their prognosis.

Against this background, the timing for the transition of patients with childhood-onset type 1 diabetes from pediatric to adult care has become a subject of much debate in recent years, which has led to a position statement being released by the ADA Transition Working Group [16], and some transition tools being offered on the National Diabetes Education Program website [17]. The Endocrine Society also launched the Transition of Care Task Force offering a toolkit for young adults with hormonal conditions and diseases including type 1 diabetes to help them transition from pediatric to adult care [18]. Despite these initiatives, evidence is limited on the transition of patients with childhood-onset type 1 diabetes from pediatric to adult care [13] and is often based on evidence from small study populations and lacking longitudinal data. Therefore, the issues and challenges associated with their transition as well as their solutions remain to be explored. Moreover, the relationship between their transition to adult care and their prognosis has not been explored.

The aim of this study is to follow up patients with childhood-onset type 1 diabetes for a maximum of 40 years to examine the timing of their transition from pediatric to adult care and the factors that contributed to their transition as well as to explore whether receiving pediatric care or adult care was associated with mortality in these patients.

## Materials and Methods

### Subjects

The study subjects were selected from the 1,384 patients with childhood-onset type 1 diabetes that compose the Japanese cohort of the Diabetes Epidemiology Research International (DERI) mortality study. The DERI mortality study is an on-going follow-up study launched in 1986 to examine the mortality status of type 1 diabetes among the four diverse population-based cohorts from Finland, USA, Israel, and Japan [19, 20]. The Japanese DERI cohort was identified through two nationwide surveys conducted in 1970 and 1981 [21, 22]. This cohort had a maximum follow-up duration of 40 years and a maximum duration of diabetes of 45 years. The representativeness of the cohort to the target population is discussed elsewhere [19,20,23]. The case ascertainment rate for the cohort was estimated to be 75% based on the reported incidence rate of type 1 diabetes (0.8 per 100,000 person-years) during that period.

As pediatric care primarily covers children less than 16 years of age in Japan, those given their diagnoses at older than 15 years were excluded in this study. Inclusion criteria were as follows: 1) diabetes diagnosed at less than 15 years of age; 2) initiation of insulin therapy within 1 month of disease onset; and 3) receiving a diagnosis between 1965 and 1969 and confirmed to be alive as of January 1, 1970, or given a diagnosis between 1970 and 1979 and confirmed to be alive as of January 1, 1980. All patients with diabetes secondary to other causes, such as Down’s syndrome or steroid treatment, were excluded. The study included a total of 1,299 patients (525 males/774 females) from the Japanese DERI cohort. Of the 1,299 patients, 1,232 were available for follow-up with a 94.8% ascertainment rate.

### Variables related to transition from pediatric to adult care

Patients were surveyed about their living status and classified as having received either pediatric or adult care at the age of 15 years. In addition, for those who were confirmed to have received pediatric care, the timing for their transition to adult care, as well as factors such as age at diagnosis, sex, calendar year of diagnosis, and population size of cities where their physicians’ offices were located when the patients were 15 years old, which may have affected their transition, were assessed as of January 1^st^, 2010 by the Kaplan-Meier method and Cox proportional hazard analyses.

Patients were also classified as having received either pediatric or adult care at the age of 30, when they were generally assumed to be independent both financially and socially, and findings were compared for differences in mortality associated with the specialty of their attending physicians (pediatricians or internists) at a patient age of 15 and 30, respectively, by using the log-rank test.

### Methods of follow-up

Patients’ attending physicians and affiliations were determined through questionnaire surveys conducted every 5 years. Their living status as of January 1^st^, 2010 was confirmed mainly through their attending physicians and the census register with the permission of the Ministry of Justice of Japan. For all patients whose date of transition remained unknown (i.e., those identified as having obviously transitioned from pediatric to adult care but without having been recorded as such), the date of their transition was defined as the median date between the last date they were confirmed to have consulted their pediatricians and the first date they were confirmed to have consulted their internists.

### Statistical analysis

All statistical analyses were performed by using SAS version 9.4 (SAS institute, Inc., Cary, North Carolina, USA). A *P* value of less than 0.05 was considered to be statistically significant (two-tailed test). The 95% confidence intervals (CIs) were calculated by using the Poisson distribution [24]. As this study used anonymized patient data obtained in routine clinical settings, informed consent was not obtained from the patients. The study was approved by the Institutional Review Board of Jikei University School of Medicine [the approval number: 17-087(4508)] and was carried out in accordance with the Declaration of Helsinki. Patient records were anonymized and de-identified prior to analysis.

## Results

### Clinical characteristics at the age of 15 years

The attending physicians were confirmed for a total of 1,093 patients (84.1%; 448 males and 645 females) but were unknown for 202 patients (76 males and 126 females), with 4 patients (1 male and 3 females) confirmed dead at the age of 15. Of these, 43.8% (*n* = 569) and 40.3% (*n* = 524) received pediatric care and adult care, respectively, at the age of 15 (Table 1).

**Table 1:** Demographics of patients with childhood-onset type 1 diabetes at the age of 15 according to the specialty of their attending physicians.

	Overall	Pediatricians	Internists	*P* value
Number of patients	1,093	569	524	N/A
Number of females	645 (59.0%)	325 (57.1%)	320 (61.1%)	0.172*
Age at diagnosis (years)	7.9 ± 3.7	7.9 ± 3.7	8.0 ± 3.8	0.524†
Calendar year of diagnosis	1975.3 ± 3.7	1975.6 ± 3.5	1975.1 ± 3.9	0.048†
Duration of diabetes at age 15 (years)	7.1 ± 3.7	7.1 ± 3.7	7.0 ± 3.8	0.524†

All data are expressed as mean ± SD.

* *P* value is calculated using chi-square test.

†*P* value is calculated using *t* test.

Compared to those receiving adult care at the age of 15, those receiving pediatric care were significantly older in regard to their calendar year of diagnosis (*P* = 0.048, *t* test), while there was no significant difference in regard to their age at diagnosis (*P* = 0.524, *t* test) and duration of diabetes at the age of 15 (*P* = 0.524, *t* test) (Table 1). There was no significant patient sex difference in the ratio of their attending physicians (pediatricians or internists) at the age of 15 (*P* = 0.172, chi-square test) (Table 1).

### Age at transition

**Fig 1:**
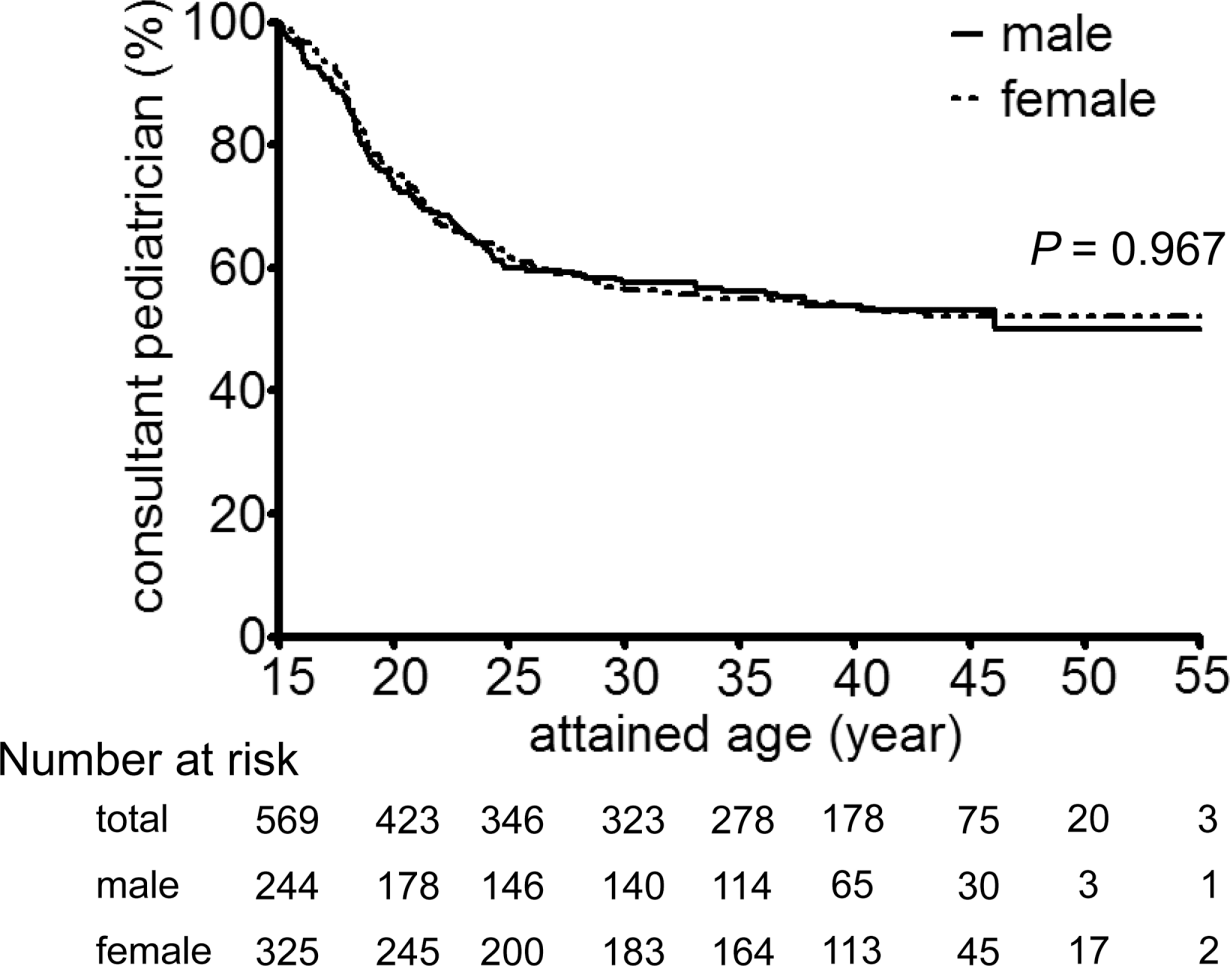
Transition from pediatric to adult care among 569 patients receiving pediatric care at the age of 15 as stratified by sex. *P*-value was calculated by log-rank test. Less than 50% of patients who had received pediatric care at the age of 15 transitioned to adult care by the age of 30, with very few transitioning to adult care after that. There was no sex difference in the timing of their transition from pediatric to adult care (*P* = 0.967, log-rank test).

Of the 569 patients receiving pediatric care at the age of 15 years, 74.2% (*n* = 423), 56.6% (*n* = 323), 53.4% (*n* = 178), and 51.3% (*n* = 20) continued to receive pediatric care at 20, 30, 40, and 50 years of age, respectively (Kaplan-Meier method, Fig 1). Less than 50% of patients who had received pediatric care at the age of 15 transitioned to adult care by the age of 30, with very few transitioning to adult care after that. There was no sex difference in the timing of transition from pediatric to adult diabetes care (*P* = 0.967, log-rank test). Their mean attained age was 42.6 ± 5.2 years as of January 1, 2010, when 71.7% (*n* = 790) were receiving adult diabetes care (including those who died after transition from pediatric to adult diabetes care), 22.9% (*n* = 252) were receiving pediatric care, with the status of care unknown in 5.4% (*n* = 59). Of the 569 patients, 266 (46.7%) transitioned to adult care, with the mean age at transition to adult care being 26.4 ± 10.8 years.

Cox proportional hazard analyses for patients with childhood-onset type 1 diabetes indicated that the younger their age at diagnosis and the smaller the population size (less than 500,000) of the cities where their attending physician’s offices were located when they were 15 years old, the significantly more likely they were to continue to receive pediatric care at the end of the study [hazard ratios (95% CI) = 0.936 (0.906, 0.968), 0.718 (0.555, 0.928), *P* < 0.001, = 0.012] (Table 2).

**Table 2:** Cox proportional hazard analyses of independent variables predicting continued consultation with pediatricians at the end of the survey.

Variable	Hazard ratio	95%CI	*P* value
Age at diagnosis (/year)	0.936	0.906–0.968	<0.001
Calendar year of diagnosis (/year)	0.978	0.946–1.010	0.181
Sex (male versus female)	0.953	0.746–1.218	0.700
Population size of the city where physicians’ offices were located at age 15:			
≥ 500,000 versus < 500,000	0.718	0.555–0.928	0.012

### Cumulative survival rate

**Fig 2:**
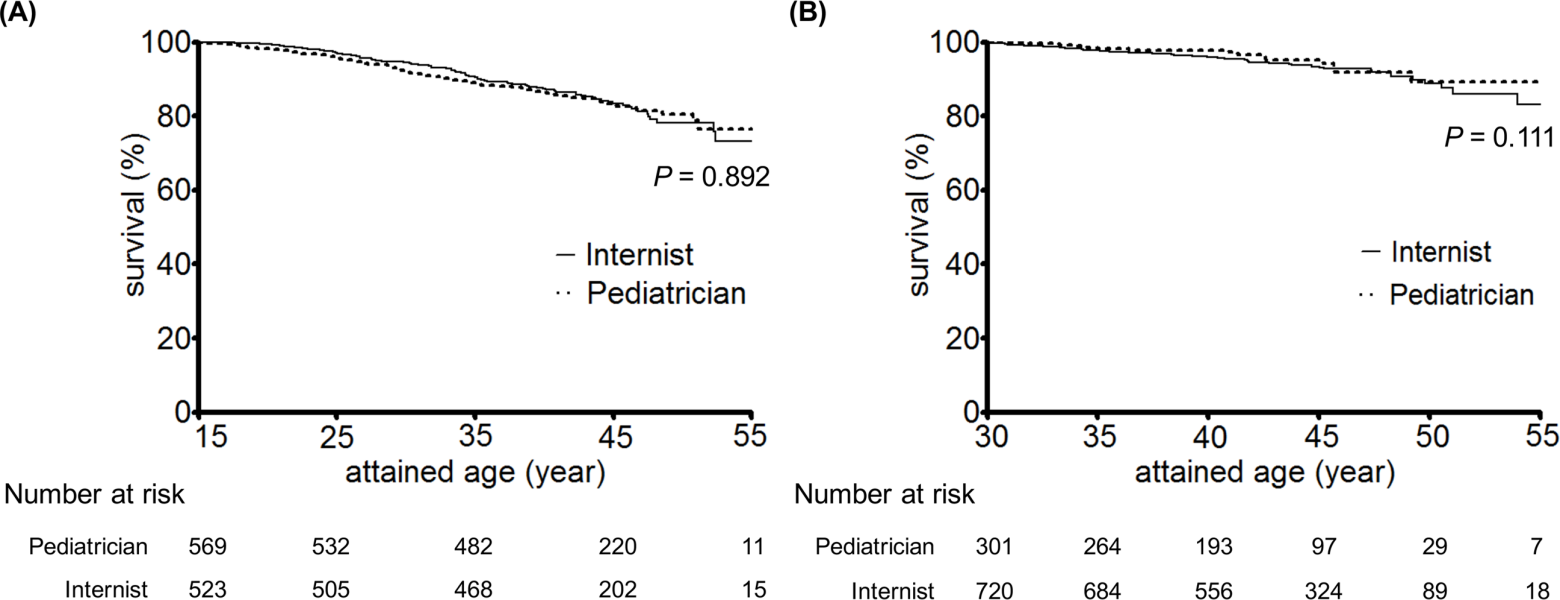
**The cumulative survival rates for the patients at the age of 15 (A) and 30 years (B) as stratified by specialty of their attending physician.** *P*-value was calculated by log-rank test.

The cumulative survival rates among the patients with childhood-onset type 1 diabetes as stratified by the type of attending physicians (pediatricians/internists) at the patient age of 15 were 100/100%, 95.5/96.9%, 90.8/92.1%, 87.4/87.1%, and 83.2/76.1% at the patient age of 15, 25, 35, 45 and 55, respectively (Fig 2A). A log-rank test revealed that the type of attending physician (pediatricians or internists) at the patient age of 15 had no significant impact on their survival (*P* = 0.892).

The cumulative survival rates among the patients at the age of 30 with childhood-onset type 1 diabetes as stratified by the type of attending physician (pediatricians/internists) were 100/100%, 98.2/97.8%, 94.3/93.4%, and 89.3/83.1% at the age of 30, 35, 45 and 55, respectively (Fig 2B). Consequently, the type of attending physician (pediatricians or internists) at the patient age of 30 had no impact on survival either (*P* = 0.111, log-rank test).

## Discussion

The Transitions Clinical Report Authoring Group consisting of the American Academy of Pediatrics, the American Academy of Family Physicians, and the American College of Physicians announced that a well-timed transition from pediatric to adult care ideally occurs between the ages of 18 and 21 years [25]. It has been reported in the USA that 77% of patients leave pediatric care by age 21 [26], but in contrast, the current study found that only 28.8% of patients who received pediatric care at the age of 15 left pediatric care by age 21, and more than 50% of these patients remained with their pediatricians even after 30 to 50 years of age.

One of the factors hampering the transition of type 1 diabetic patients from pediatric to adult care in Japan is the incidence of type 1 diabetes in the country, which is much lower than that reported in the USA or European countries [27]. This may imply that even when patients with type 1 diabetes seek adult care, their physicians may remain relatively less familiar with their disease and therefore less well experienced in treating these patients or may have difficulty referring such patients to any other appropriate physician and that, as a consequence, these patients may be more likely to continue to consult their pediatricians without ever transitioning to adult care. Our study shows that the younger the age at diagnosis and the earlier the calendar year of their diagnosis, the significantly more likely patients were to continue to receive pediatric care, even after reaching adulthood, which supports the above-mentioned view. Given that the childhood onset of their disease was shown to be a factor strengthening the relationships between the patients, their family, and their attending physicians, this may have contributed to their continued consultation with their pediatricians, leading to reluctance for them to transition from pediatric to adult care. In addition, generally, less populated areas tend to be less medically equipped and this may make it difficult for any patient in such an area to find the next appropriate attending physician. Our findings suggest that this may have led them to continue with pediatric care without transitioning to adult care.

Another factor hampering the transition of type 1 diabetic patients from pediatric to adult care in Japan may be found in the current state of the healthcare system in the country, where medical consultation will be made available even to adult patients without a referral form or prior appointment at most major hospitals. This implies that adult care physicians, particularly diabetologists, may be obliged to see a large number of outpatients on a daily basis as they present to them, and the consultation time for each patient is likely to be shorter and the time on the waiting list longer than when they would consult their pediatricians. This may be among the factors contributing to these patients continuing with pediatric care even after reaching adulthood in Japan. Therefore, putting systems in place within healthcare that allow for a gradual transition from pediatric to adult care may help bridge the current gap between these stages of a patient’s treatment.

Our study findings also suggest that the type of attending physician for the patient at ages of both 15 and 30 was not a prognostic factor for mortality in patients with childhood-onset type 1 diabetes. During the periods covered by the study, both pediatric care and adult care appears to have provided similar medical care for patients with type 1 diabetes in Japan. However, their mean attained age (42.6 ± 5.2 years as of January 1, 2010) suggests that they were reaching a point where they may be more susceptible to lifestyle-related diseases, such as cancer and cardiovascular diseases, and may require specialist care for both type 1 diabetes and lifestyle-related diseases. Indeed, cardiovascular disease is shown to be a leading cause of death among Japanese patients with childhood-onset type 1 diabetes of more than 20 years’ duration [1], although at least 65% of the patients who died of cardiovascular disease had been receiving dialysis. In these patients, therefore, preventive measures against atherosclerosis and lifestyle-related diseases need to be implemented. Further study is required to determine if the type of attending physician (pediatrician or internist) may be among the factors predicting mortality.

To the best of our knowledge, this is the first study to describe the timing of transition from pediatric to adult care among patients with childhood-onset type 1 diabetes and associated factors, as well as the relationship between their attending physicians (pediatricians or internists) and their prognosis.

The current study has several limitations. First, this study was conducted in Japan and may not be applicable to other countries. Thus, a similar study conducted in any other country may yield different results than ours, depending not only on the healthcare system but also the ethnic groups and races involved. Second, patients’ individual data, such as their glycemic control, diabetic complications, and frequency of visits, were not included in the analysis. The level of glucose control, status of diabetic complications, and frequency of visits may have affected their mortality. In addition, among those who had already consulted internists at the age of 15, some may have transitioned from pediatric to adult care earlier for their advanced diabetic complications. Again, further research is thus needed to clarify the factors contributing to their early transition to adult care.

Currently, there is no well-established evidence-based clinical guidelines in Japan for patients with childhood-onset type 1 diabetes transitioning from pediatric to adult care. Study results suggest that the timing of their transition does not affect their prognosis as of January 1, 2010 when they had a mean attained age of 42.6 ± 5.2 years and therefore that it may not be necessary to set strict age limits for their transition from pediatric to adult care. Thus, flexible transition may be promoted, with consideration given to the medical environment each patient finds himself or herself in, their developmental stage, and self-care abilities, or with a transition phase put in place to allow these patients the choice of consulting either pediatricians or internists. However, given that over 50% of patients continuing with pediatric care at the age of 30 are less likely to transition to adult care, it may be necessary to set an upper age limit for their transition in view of lifestyle-related diseases requiring comprehensive care in these patients. Further study is required to establish the guidelines recommending the best timing for transition to adult care for individual patients in Japan.

## Supporting Information

S1 Dataset The primary dataset of the current study. (XLSX)
